# *Pharmaceuticals* Best Paper Award 2015

**DOI:** 10.3390/ph8020277

**Published:** 2015-06-05

**Authors:** Jean Jacques Vanden Eynde

**Affiliations:** Laboratory of Organic Chemistry (FS), University of Mons-UMONS, Place du parc, 23, 7000 Mons, Belgium; E-Mail: jean-jacques.vandeneynde@ex.umons.ac.be

It is always a difficult task for a jury to classify articles from a selection of outstanding manuscripts. This was the case again this year when we had to award a prize to three contributions only, whereas we read so many interesting and exciting results published in 2012 and 2013. Anyway, the members of the jury did their job and have the pleasure to announce that the three following publications won the *Pharmaceuticals* Best Paper Award for 2015.

1st Prize
  **Ivana Cacciatore, Erika Fornasari, Leonardo Baldassarre, Catia Cornacchia, Stefania Fulle, Ester Sara Di Filippo, Tiziana Pietrangelo and Francesco Pinnen**
  A Potent (R)-alpha-bis-lipoyl Derivative Containing 8-Hydroxyquinoline Scaffold: Synthesis and Biological Evaluation of Its Neuroprotective Capabilities in SH-SY5Y Human Neuroblastoma Cells
  *Pharmaceuticals* **2013**, *6*(1), 54-69; doi:10.3390/ph6010054
  Available online: http://www.mdpi.com/1424-8247/6/1/54
2nd Prize
  **Albert J. Chang, Ravindra DeSilva, Sandeep Jain, Kimberley Lears, Buck Rogers and Suzanne Lapi**
  89Zr-Radiolabeled Trastuzumab Imaging in Orthotopic and Metastatic Breast Tumors
  *Pharmaceuticals* **2012**, *5*(1), 79-93; doi:10.3390/ph5010079
  Available online: http://www.mdpi.com/1424-8247/5/1/79
3rd Prize
  **Luiza I. Hernandez, Katie S. Flenker, Frank J. Hernandez, Aloysius J. Klingelhutz, James O. McNamara and Paloma H. Giangrande**
  Methods for Evaluating Cell-Specific, Cell-Internalizing RNA Aptamers
  *Pharmaceuticals* **2013**, *6*(3), 295-319; doi:10.3390/ph6030295
  Available online: http://www.mdpi.com/1424-8247/6/3/295

The Prize Awarding Committee describes the article “A Potent (R)-alpha-bis-lipoyl Derivative Containing 8-Hydroxyquinoline Scaffold: Synthesis and Biological Evaluation of Its Neuroprotective Capabilities in SH-SY5Y Human Neuroblastoma Cells” as “An interesting preclinical approach, newly combining antioxidant/metal-chealting (in neuroprotective) molecules for the treatment of neurodegenerative diseases, such as PD or AD”.

We believe these three papers represent valuable contributions to *Pharmaceuticals* and the scientific literature. On behalf of the Prize Awarding Committee and the Editorial Board of *Pharmaceuticals*, we warmly congratulate these three groups. In recognition of their accomplishment, Dr. Ivana Cacciatore, Dr. Suzanne Lapi, and Dr. Paloma H. Giangrande will have the opportunity of publishing an additional Open Access format review or research paper, free of charge, in our Journal, *Pharmaceuticals*.

*Prize Awarding Committee*

Editor-in-Chief
  **Dr. Jean Jacques Vanden Eynde**
  Laboratory of Organic Chemistry (FS), University of Mons-UMONS, Place du parc, 23, 7000 Mons, Belgium
  E-Mail: jean-jacques.vandeneynde@ex.umons.ac.be
Associate Editor
  **Dr. Annie Mayence**
  Formerly Professor at the Haute Ecole Provinciale de Hainaut-Condorcet, 7330 Saint-Ghislain, Belgium
  E-Mail: annie.mayence@condorcet.be
Editorial Board Member
  **Prof. Dr. Klaus Kopka** Division of Radiopharmaceutical Chemistry, Research Program Imaging and Radiooncology, German Cancer Research Center (dkfz), Im Neuenheimer Feld 280, D-69120 Heidelberg, Germany
  E-Mail: k.kopka@dkfz-heidelberg.de
Editorial Board Member
  **Prof. Dr. Thomas Rades** Reseach Chair in Pharmaceutical Design and Drug Delivery Faculty of Health and Medical Sciences, Department of Pharmacy, University of Copenhagen, Universitetsparken 2, 2100 København Ø, Denmark
  E-Mail: thomas.rades@sund.ku.dk

